# Multiple myeloma incidence and mortality trends in the United States, 1999–2020

**DOI:** 10.1038/s41598-024-65590-4

**Published:** 2024-06-24

**Authors:** David T. Zhu, Andrew Park, Alan Lai, Lingxiao Zhang, Hiba Attar, Timothy R. Rebbeck

**Affiliations:** 1 Medical Scientist Training Program, School of Medicine, Virginia Commonwealth University, Richmond, VA 23298 USA; 2 School of Medicine, Virginia Commonwealth University, 1201 E Marshall St, RichmondRichmond, VA 23298 USA; 3 Department of Chronic Disease Epidemiology, Yale School of Public Health, New Haven, CT 06510 USA; 4 Department of Microbiology and Immunology, Schulich School of Medicine & Dentistry, London, ON N6A 5C1 Canada; 5 Dana-Farber Cancer Institute and Harvard TH Chan School of Public Health, Boston, MA 02215 USA

**Keywords:** Multiple myeloma, Incidence, Mortality, Cancer disparities, Racial and ethnic disparities, Health care, Oncology

## Abstract

Multiple myeloma (MM) is a plasma cell disorder accounting for approximately 10% of hematologic malignancies. There is limited epidemiological evidence regarding the long-term trends and disparities in MM in the US. We conducted a multiple time point cross-sectional study using MM incidence rate data from the Surveillance, Epidemiology, and End Results (SEER) database and mortality data from the CDC Wide-Ranging Online Data for Epidemiologic Research (CDC WONDER) Underlying Cause of Death database between 1999 and 2020. During this period, MM incidence has steadily increased, while MM mortality has steadily decreased, with substantial racial and ethnic disparities. Non-Hispanic Black individuals exhibited the highest incidence rates, which consistently rose from 12.02 (95% CI 10.54, 13.64) in 1999 to 14.20 (95% CI 12.93, 15.55) per 100,000 population by 2020. Non-Hispanic American Indian/Native Alaskans and Asian/Pacific Islanders demonstrated the lowest incidence rates of 5.59 (95% CI 2.69, 10.04) and 3.56 (95% CI 2.94, 4.27) per 100,000 population in 1999 to 5.76 (95% CI 3.49, 8.90) and 3.92 (95% CI 3.46, 4.42) per 100,000 population, respectively, by 2020. Disparities by gender, age, US census region, and rurality were observed, underscoring the importance of targeted, equity-centered interventions and MM screening initiatives for at-risk populations.

## Introduction

Multiple myeloma (MM) is a clonal plasma cell proliferative disorder with abnormally elevated serum monoclonal immunoglobulins, often resulting in severe end-organ damage when left untreated^1^. Clinical manifestations may include hypercalcemia, renal failure, anemia, and bone disease^2^. Diagnostic criteria typically include ≥ 10% clonal plasma cells in bone marrow, demonstrable end-organ damage, and specific myeloma-defining biomarkers^3,4^. Notably, MM has one of the lengthiest diagnostic intervals among cancers, largely due to its rarity and the presence of nonspecific symptoms such as back and bone pain, often leading to diagnosis through incidental findings^5^.

MM accounts for approximately 2% of cancer diagnoses and 10% of hematologic malignancies in the United States, notably, with incidence rates rising by 40% in the United States and nearly 130% globally since 1990^6,7^. During this period, MM mortality rates have fallen to 18% while five-year overall survival rates have increased to nearly 54%, driven by novel therapies introduced over the past two decades amidst other advancements in cancer care^7,8^. Nevertheless, MM remains a significant cause of mortality and morbidity, leading to 2.1 million disability-adjusted life-years lost since 2016^9,10^. Significant disparities exist, with incidence rates among non-Hispanic Black Americans being more than two-fold higher compared to their non-Hispanic White counterparts, as well as disproportionately higher five-year mortality rates^7^. In contrast, Asian American and Pacific Islander (AAPI) individuals consistently demonstrated the lowest rates of MM incidence and mortality^7^.

Monoclonal gammopathy of undetermined significance (MGUS) and smoldering multiple myeloma (SMM) are established precursors to MM. Thus, early detection and intervention of MGUS and SMM are crucial to slow progression to MM, improve life expectancy, and mitigate potential end-organ damage^11^. Prior research suggests that patients previously diagnosed with MGUS have a lower likelihood of presenting with end-organ disease at MM diagnosis, correlating with a 13–15% increase in their overall survival, possibly attributed to their access to earlier treatment and support services^10,12,13^. Current consensus guidelines recommend continuous monitoring of individuals diagnosed with MGUS to identify progression to MM, yet the asymptomatic nature of MGUS and SMM poses challenges for early clinical intervention^14^. This highlights the need for improved screening for MGUS, SMM, and MM among high-risk subpopulations such as Black Americans, elderly patients, patients with obesity, and others to improve prognosis^15,16^.

Further, social determinants such as socioeconomic status, access to healthcare, education, employment, and community factors can dramatically improve prognosis and survival outcomes in MM patients^17^. Social factors such as equitable access to care, including systemic actions taken to remove financial barriers, are crucial to ensure that vulnerable populations have access to novel therapeutic agents and supportive therapies^18^. For instance, dietary interventions, including meals comprised of whole food plant-based meals, have also been associated with improvements in metabolic and microbiome biomarkers of MGUS and SMM progression, underscoring the importance of improving access to nutritious food options for MM patients living within food deserts^19^.

To address the limited understanding of the features that may drive MM screening, this study aims to (1) comprehensively describe geospatial and temporal trends in MM incidence and mortality over the past two decades, and (2) identify racial and ethnic disparities in MM incidence and mortality by population-level sociodemographic and spatial characteristics.

## Methods

In this serial cross-sectional study between 1999 and 2020, we obtained MM incidence data from the Surveillance, Epidemiology, and End Results (SEER) 12 database and MM mortality data from the CDC Wide-Ranging Online Data for Epidemiologic Research (CDC WONDER) Underlying Cause of Death database. Self-identified race/ethnicity (SIRE) data were obtained from these data sources. Delayed-adjusted incidence rates within the SEER 12 database allowed for a standard delay period of 22 months, accounting for updates such as new cases or corrections from previous reported cases^20^. Age-adjusted mortality rates within the CDC WONDER database were determined using the direct method, utilizing the age distribution of the U.S. standard population^21^. Delayed-adjusted incidence rate data were examined both overall and stratified by sex and age. Death certificates for decedents with ICD-10 codes for multiple myeloma and malignant plasma cell neoplasms (C90) as underlying causes of death were examined for age-adjusted mortality rates.

We further examined mortality rates stratified by sex (male vs. female), age, self-identified racial and ethnic (SIRE) groups, US census region, and urbanicity. We defined the following age groups: individuals aged 39 years or below, followed by five-year intervals within the range of 40 to 84 years, and finally individuals aged 85 years or above. Further, we defined the following SIRE groups: Hispanic individuals as well as non-Hispanic White, Black, American Indian/Alaskan Native (AIAN), and AAPI individuals. Notably, the non-Hispanic AIAN population within the SEER 12 database also encompasses individuals residing within a Purchased/Referred Care Delivery Area (PRCDA)^22^. We analyzed four US census regions (Northeast, Midwest, South, and West) along with urbanization status, classified according to the 2013 NCHS Urban–Rural Classification Scheme for Counties (non-core, micropolitan, small metro, medium metro, large fringe metro, and large central metro)^21^.

Temporal trends were plotted using R (version 4.3.0) and Graphpad PRISM (version 10.2.1). Due to the de-identified and publicly available nature of our data, it was determined by the Virginia Commonwealth University School of Medicine that this study does not constitute human subjects research, and thus was exempt from institutional review.

## Results

Between 1999 and 2020, a total of 53,527 MM cases were identified across 12 registries in the US from the SEER database, with 33,051 (61.75%) non-Hispanic White, 8,516 (15.91%) non-Hispanic Black, 4,516 (8.44%) non-Hispanic AAPI, 433 (0.81%) AIAN, and 6,835 (12.77%) Hispanic individuals. During this same period, 252,005 MM decedents were identified from the CDC WONDER database, with 186,629 (74.06%) non-Hispanic White, 43,858 (17.40%) non-Hispanic Black, 4,682 (1.86%) non-Hispanic AAPI, 1,155 (0.46%) non-Hispanic AIAN, and 15,681 (6.22%) Hispanic decedents.

**Figure 1:**
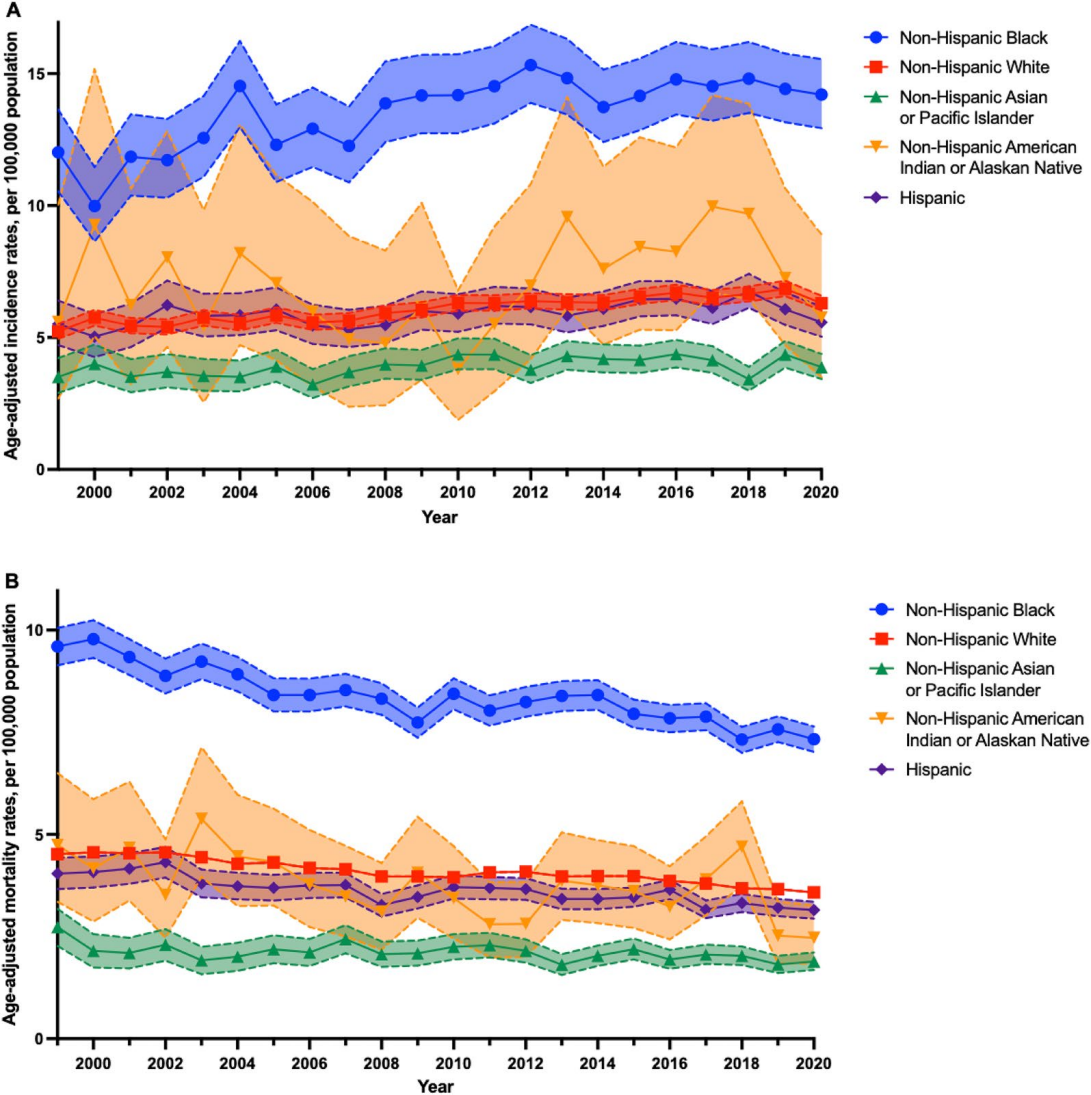
Racial and ethnic differences in multiple myeloma incidence (**A**) and mortality (**B**) between 1999 and 2020. Age-adjusted incidence rates (95% CI) of multiple myeloma by race and ethnicity between 1999 and 2020, as obtained from SEER 12 (**A**). Age-adjusted mortality rates (95% CI) of multiple myeloma by race and ethnicity between 1999 and 2020, as obtained from CDC WONDER (**B**). All figures were generated using R (version 4.3.0) and Graphpad PRISM (version 10.2.1).

Age-adjusted MM incidence rates slightly increased among all five SIRE groups from 1999 to 2020 (Fig. 1). Notably, non-Hispanic Black individuals consistently experienced the highest incidence rates between 1999 to 2020, rising from 12.02 (95% CI 10.54, 13.64) in 1999 to 14.20 (95% CI 12.93, 15.55) per 100,000 population in 2020. Non-Hispanic AIAN individuals and AAPI individuals, in comparison, exhibited the lowest incidence rates, which rose from 5.59 (95% CI 2.69, 10.04) and 3.56 (95% CI 2.94, 4.27) per 100,000 population, respectively, in 1999 to 5.76 (95% CI 3.49, 8.90) and 3.92 (95% CI 3.46, 4.42) per 100,000 population, respectively, by 2020.

Additionally, consistent declines in MM mortality were observed between 1999 and 2020 in all SIRE groups (Fig. 1). Non-Hispanic Black individuals consistently exhibited the highest mortality rates throughout the study period, decreasing from 9.60 (95% CI 9.14, 10.05) to 7.33 (95% CI 7.02, 7.64) per 100,000 from 1999 to 2020. In contrast, non-Hispanic AIAN and AAPI individuals experienced the lowest mortality rates during the same timeframe, with MM mortality rates declining from 4.74 (95% CI 3.35, 6.50) to 2.47 (95% CI 2.27, 3.18) per 100,000 population among non-Hispanic AIAN individuals, and decreasing from 2.73 (95% CI 2.27, 3.18) to 1.89 (95% CI 1.68, 2.11) per 100,000 population among AAPI individuals.

**Figure 2:**
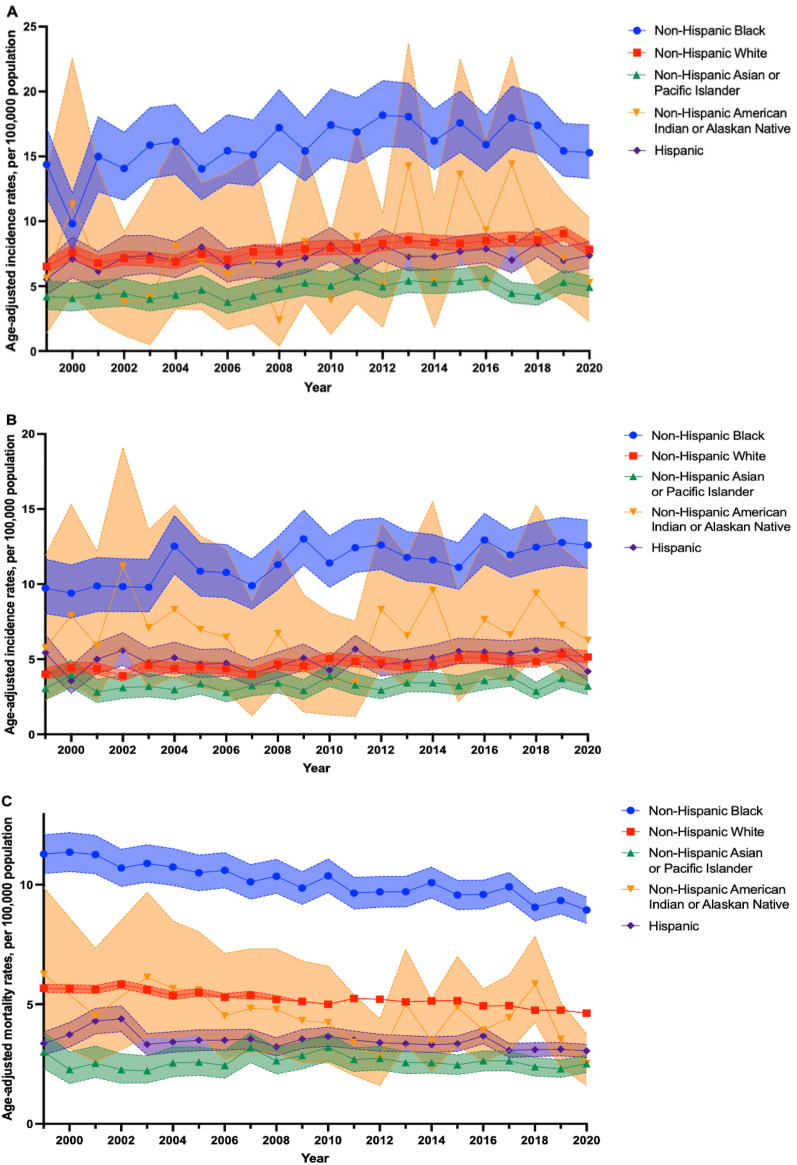

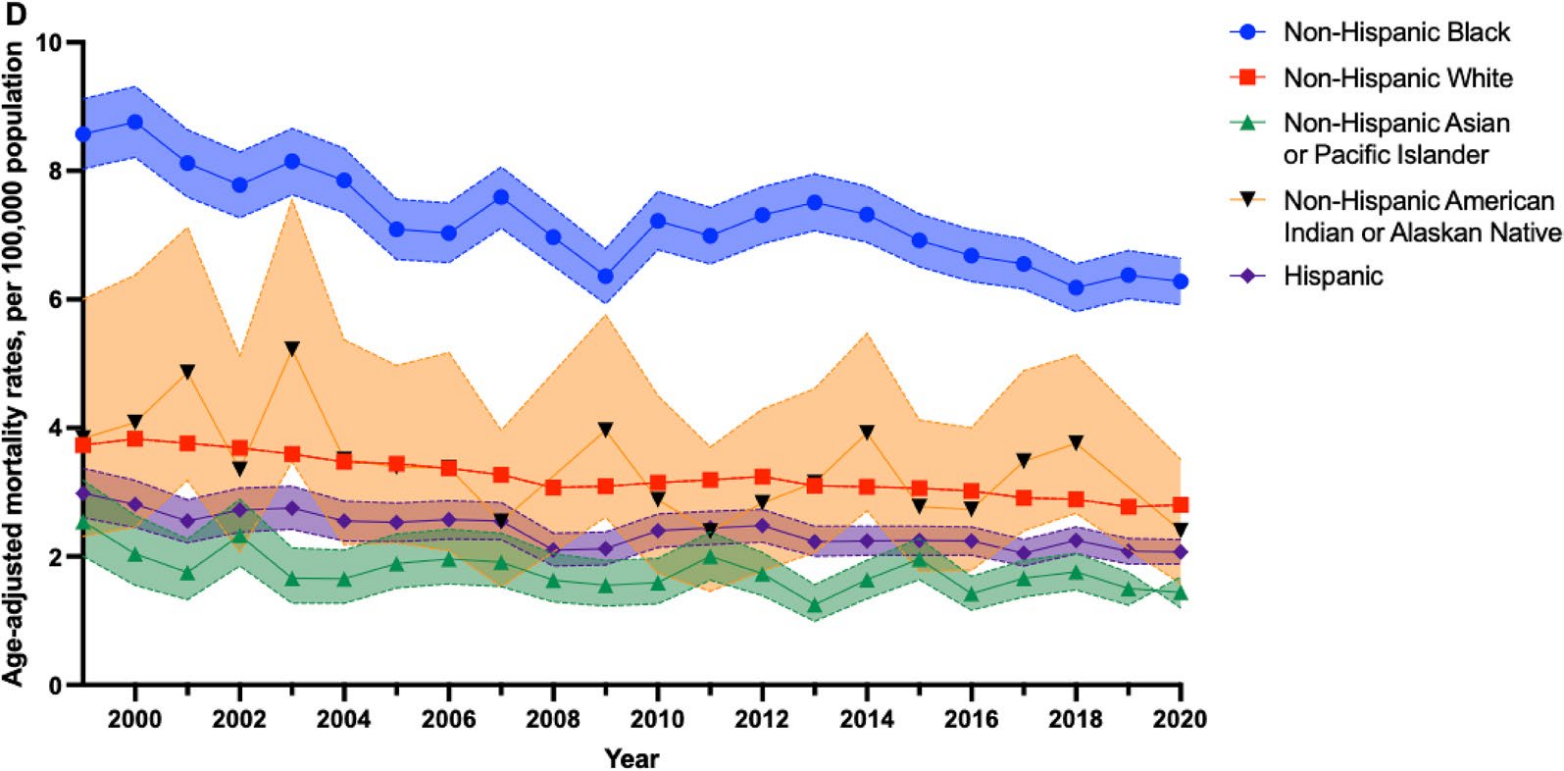
Racial and ethnic differences in multiple myeloma incidence among males (**A**) and females (**B**), and mortality among males (**C**) and females (**D**), between 1999 and 2020. Age-adjusted incidence rates (95% CI) of multiple myeloma by race and ethnicity between 1999 and 2020, as obtained from SEER 12, for males (**A**) and females (**B**). Age-adjusted mortality rates (95% CI) of multiple myeloma by race and ethnicity between 1999 and 2020, as obtained from CDC WONDER, for males (**C**) and females (**D**). All figures were generated using R (version 4.3.0) and Graphpad PRISM (version 10.2.1).

For both men and women, MM incidence and mortality rates in non-Hispanic Black individuals were consistently higher compared to other SIRE groups, with men exhibiting higher rates than women. Notably, MM incidence from 1999 to 2020 rose from 14.74 (95% CI 12.18, 17.64) to 15.92 (95% CI 13.86, 18.18) per 100,000 population among non-Hispanic Black men, compared to the increase from 9.97 (95% CI 8.24, 11.94) to 12.99 (95% CI 11.41, 14.74) per 100,000 population among non-Hispanic Black women (Fig. 2). Similarly, despite MM mortality rates steadily declining for non-Hispanic Black individuals, these rates remained persistently higher than other SIRE groups for both men and women (Fig. 2).

MM mortality rates from 1999 to 2020 decreased from 11.28 (95% CI 10.47, 12.09) to 8.94 (95% CI 8.39, 9.48) per 100,000 population among non-Hispanic Black men, while decreasing from 8.57 (95% CI 8.03, 9.12) to 6.28 (95% CI 5.92, 6.64) per 100,000 population among non-Hispanic Black women. Non-Hispanic AIAN and AAPI individuals exhibited the lowest MM incidence and mortality rates, with rates among men consistently surpassing women. MM incidence rates consistently declined for non-Hispanic AIAN and AAPI men between 1999 (AIAN: 5.61 [95% CI 4.34, 6.95] per 100,000; AAPI: 5.26 [95% CI 2.27, 10.25] per 100,000) and 2020 (AIAN: 5.26 [95% CI 2.27, 10.25] per 100,000; AAPI: 4.93 [95% CI 4.16, 5.79] per 100,000) (Fig. 2). Contrary to men, MM incidence rates increased from 1999 to 2020 among non-Hispanic AIAN and AAPI women, increasing from 1999 (AIAN: 5.77 [95% CI 2.27, 11.88) per 100,000; AAPI: 3.05 [95% CI 2.29, 3.98] per 100,000) to 2020 (AIAN: 6.26 [95% CI 3.15, 11.01] per 100,000; AAPI: 3.21 [95% CI 2.65, 3.85] per 100,000) (Fig. 2). Further, MM mortality rates among non-Hispanic AIAN and AAPI men decreased from 1999 (AIAN: 6.25 [95% CI 3.71, 9.88] per 100,000; AAPI: 3.00 [95% CI 2.31, 3.83] per 100,000) to 2020 (AIAN: 2.52 [95% CI 1.61, 3.75] per 100,000; AAPI: 2.51 [95% CI 2.14, 2.88] per 100,000) (Fig. 2). Similarly, MM mortality rates declined for non-Hispanic AIAN and AAPI women between 1999 (AIAN: 3.84 [95% CI 2.31, 6.00] per 100,000; AAPI: 2.54 [95% CI 2.00, 3.18] per 100,000) and 2020 (AIAN: 2.40 [95% CI 1.57, 3.51] per 100,000; AAPI: 1.44 [95% CI 1.20, 1.68] per 100,000) (Fig. 2).

Disparities in age-specific MM incidence and mortality also changed with advancing age. Compared to non-Hispanic White individuals (reference), age-specific MM incidence and mortality rates were consistently higher among Non-Hispanic Black individuals, while lower rates were noted among non-Hispanic AAPI individuals (Supplementary Tables 1–2). Notably, compared to non-Hispanic White individuals, the highest incidence rate ratio (IRR) was observed among non-Hispanic Black individuals aged 50 to 54 years (IRR: 3.34), while the lowest rate ratio was noted among non-Hispanic AAPI individuals aged 85 years and older (IRR: 0.36) (Supplementary Table 1). Notably, in the non-Hispanic AIAN population, incidence rates peaked at 34.55 (11.22, 78.68) per 100,000 among those aged 65 to 69 years, then dropped by nearly half to 19.10 (2.31, 65.69) per 100,000 among those aged 70 to 74 years. (Supplementary Table 1). Additionally, compared to non-Hispanic White individuals, mortality rate ratio (MRR) was highest among non-Hispanic Black individuals aged 55 to 59 years (MRR: 2.77), whereas lowest among non-Hispanic AAPI individuals aged 85 years or older (MRR: 0.43) (Supplementary Table 1).

With regard to geospatial trends, MM mortality rates were highest in the South among non-Hispanic Black (MRR: 2.22) and AIAN individuals (MRR: 0.81), while highest among non-Hispanic AAPI individuals in the South and Midwest (MRR: 0.57), and Hispanic individuals in the West (MRR: 0.70) compared to non-Hispanic White individuals (Supplementary Table 2). Additionally, MM mortality rates among non-Hispanic Black individuals were highest within small metropolitan areas (MRR: 2.59), whereas lowest within non-metropolitan areas (MRR: 2.25) (Supplementary Table 2).

## Discussion

To our knowledge, this was the first epidemiological study to comprehensively describe trends and disparities in MM incidence and mortality over the past two decades. We found that MM incidence rates rose slightly over this period, while MM mortality steadily decreased from 1999 to 2020. However, substantial disparities were apparent, wherein non-Hispanic Black populations consistently exhibited the highest incidence and mortality of MM, which persisted across strata defined by age, US census region, and urbanization status. Further, we report that non-Hispanic AIAN and AAPI individuals exhibited the lowest MM incidence and mortality rates. Men exhibited the highest MM incidence and mortality rates, highlighting gender disparities. Overall, these disparities highlight the significant burden of MM on patients and the healthcare system, underscoring the need for targeted interventions and screening initiatives for the at-risk populations identified.

Our data aligns with existing literature that non-Hispanic Black Americans exhibit the highest MM incidence and mortality rates^23–25^. These disproportionately higher rates observed among non-Hispanic Black Americans highlight the imperative for further research examining the multifaceted etiology of MM morbidity and mortality, encompassing both genetic predispositions and socio-environmental determinants to inform future targeted intervention strategies.

The higher MM mortality rates that we noted among non-Hispanic Black Americans may be partially influenced by a higher prevalence of MGUS, a precursor lesion to MM, compared to other SIRE groups, potentially explaining the elevated MM incidence rates^26–28^. The risk of progression from MGUS to MM across SIRE groups suggests that the greater incidence of MGUS in certain populations may help explain the higher rates of MM within those groups^26–28^. Specific human leukocyte antigen (HLA) alleles linked to MM susceptibility, coupled with a heightened familial predisposition to MM and related plasma cell dyscrasia, may further contribute to this racial disparity^29–31^. Additionally, structural racism experienced by Black and Hispanic Americans may exacerbate disparities in MM early detection, access to treatment, and outcomes. Prior research demonstrates Black American and Hispanic patients are more likely to experience delays in accessing treatments such as autologous stem cell transplants (ASCT) compared to White patients, hindering their ability to benefit fully from these treatments^32^.

Additionally, obesity—an established risk factor for MM and MGUS—disproportionately affects non-Hispanic Black individuals, suggesting a potential pathway through which obesity exacerbates the observed MM disparity^15,16,24^. However, while obesity plays a part in every stage of MM progression, the greatest impacts are at the level of precursor disease states like MGUS and SMM^15^. Consequently, targeted screening interventions within high-risk populations, such as those with higher obesity rates, may offer strategic avenues for earlier MM detection and intervention, thus, mitigating the burden of MM within these communities. Broader socioeconomic and structural inequalities also contribute to SIRE disparities in MM incidence and mortality. Structural barriers, including lower median incomes and higher unemployment rates among non-Hispanic Black populations, limit access to quality healthcare and novel treatments, which may contribute to variations in survival outcomes among MM patients^7,8,24,25^. Socioeconomic status may influence other upstream social factors such as education, income, and access to health insurance, all crucial for managing MM, particularly given the substantial financial burden and distress associated with its management^24,25^.

Moreover, prior research demonstrates that health literacy rates may influence the management and outcomes of MM, as higher rates have been documented to reduce emergency department visits, improve survivorship care, and help maintain or improve quality of life among MM survivors^34,35^. The discrepancy in MM mortality rates among on-Hispanic Black individuals may potentially be explained in part by health literacy challenges; even among those with adequate understanding of MM, access to care remains a complex issue, particularly for costly therapeutic strategies such as ASCT^32–35^. Prior research shows that non-Hispanic Black patients are not only less likely to undergo ASCT but also tend to initiate ASCT later in the disease course, leading to a longer interval from diagnosis to the initiation of novel therapy compared to their non-Hispanic White counterparts^35^.

The overall decline in MM mortality rates over the past two decades, consistent with prior research^8^, is encouraging. The decline in mortality rates aligns with the introduction of novel therapeutic agents boasting higher efficacy profiles and reduced toxicity, which are shown to be particularly beneficial for elderly MM patients ineligible for ASCT^36,37^. Further, advancements in ASCT have also led to improved MM prognosis and survival^37,38^. Immune-based therapies have also contributed to significant improvements in the MM treatment efficacy across diverse patient cohorts^39^. For instance, anti-CD38 monoclonal antibodies, such as daratumumab, as well as other novel MM therapeutics—including bortezomib, lenalidomide, and dexamethasone—have demonstrated prolonged median progression-free survival of 41 months compared to the 8.5 months for control cohorts^39^. Despite these promising developments, social and structural factors—such as limited access to primary care providers and cancer specialists, higher poverty rates, a greater number of uninsured patients, and underrepresentation in clinical trials—hinder Black patients from fully reaping the benefits from ASCT and novel pharmacological agents used to treat multiple myeloma^35^.

Despite these advancements, routine screening for MM is not currently a part of public health interventions and needs to be prioritized for high-risk subgroups. For instance, targeted studies such as iStopMM and PROMISE have investigated the benefits of screening in high-risk populations based on a wide array of sociodemographic factors—such as age, race, family history (e.g., African ancestry), and genetic markers—to identify at-risk individuals with MM precursor conditions such as MGUS or smoldering myeloma, and thereby improve early diagnosis and treatment^40,41^. In addition to sociodemographic factors, cytogenetic abnormalities such as del(17p), t(4;14), t(14;16), and gain(1q) can also be used to identify patients with high-risk disease at the time of diagnosis^42^. Implementing screening protocols, particularly in regions marked by heightened MM vulnerability and prevalent risk factors, is crucial for addressing the urgent need for early detection and intervention strategies concerning MM within marginalized communities and populations.

Additionally, our findings also indicate that AAPI populations exhibit the lowest rates of MM incidence and mortality compared to other ethnicities, corroborating with prior research^7,32,35^. Historically, AAPI populations have shown superior overall survival rates alongside low incidence rates in comparison to non-Hispanic white populations, as documented by several studies^7,32,35^. Factors contributing to this trend may include genetic polymorphisms, such as the NQO1*2/*2 polymorphisms observed in Koreans, as well as higher socioeconomic status among AAPI populations^7,32,35^.

The limited incidence and reporting of MM within AAPI populations may complicate future studies, particularly those aiming to disaggregate MM incidence and mortality within specific subgroups. To our knowledge, there have been no studies to date examining MM incidence and mortality amongst AAPI subgroups, warranting further in-depth investigation in relation to documented risk factors in AAPI communities such as linguistic and health literacy challenges, limited primary care physician visits, low screening rates, and more^43^.

We also observed substantial variability in age-adjusted incidence and mortality data for MM among non-Hispanic AIAN/PRCDA populations, which may be attributed to significant data suppression (due to small sample sizes) within the SEER and CDC Wonder databases. This likely results from various social and statistical factors, including the chronic underfunding of Indian Health Services (IHS) and tribal healthcare facilities, leading to insufficient cancer screening services^44^. The displacement of AIAN populations to rural areas by citizenry and US military further compounds limited healthcare access and late-stage disease detection, particularly in MM, posing challenges for obtaining essential incidence and mortality data^45^. Despite attempts to address racial misclassification by linking data to IHS patient registration, substantial misclassification of AIAN individuals persists, likely leading to an underestimation of the cancer burden^46,47^. Even with efforts to mitigate misclassification, these challenges, coupled with the rarity of MM and limited screening opportunities restricted to PRCDA counties, contribute to significant data suppression, hindering accurate analysis of incidence and mortality rates among AIAN populations^46,47^. Further efforts are needed to understand the social and structural factors contributing to the high incidence rates among vulnerable subpopulations of AIAN individuals, such as those aged 65 to 69 years. These factors may include socioeconomic status, exposure to carcinogens or environmental toxins, limited access to medical care, diagnostic delays, the prevalence of chronic conditions and multimorbidity, among others. Overall, the isolated geographic distribution, limited screening opportunities, and racial misclassification collectively limit the comprehensive analysis of AIAN data, and further research is needed to better understand the burden of MM within these communities and unique risk factors.

Geospatial analyses have also revealed regional disparities in age-adjusted MM incidence across the United States, with higher rates in the Southeast, Capital District, and New York and lower rates in the Southwest and West^48^. The spatial clustering of MM likely results from a multifaceted interplay of demographic, genetic, lifestyle, and environmental risk factors. However, our US census region-stratified data have revealed that the highest rates of MM mortality within AANHPI populations are concentrated in Western states while the lowest rates were found in the Midwest and Northeast. The differences may reflect the different compositions of AANHPI populations in different regions of the US. For example, 45% of AAPI individuals are living in Western states, with nearly 30% within California, and only 12% in the Midwest and 19% in the Northeast^49^.

Our gender-stratified analyses revealed that MM mortality and incidence rates were generally higher among men than women. These findings echo trends observed in prior studies, which consistently show that men are approximately 1.5-fold more likely to develop MM^50^. Independent of factors like age and socioeconomic status, male gender remains a risk factor for MGUS and MM^25^. Gender disparities in MM can be further explained by the increased incidence of MGUS among healthy male populations. The higher incidence of MM in males may also imply that sex plays a role in MM pathogenesis^25^, although the underlying mechanisms remain poorly understood, warranting further exploration. One plausible explanation for the higher risk among men is the greater prevalence of hyperdiploidy, characterized by the gain of multiple odd-numbered chromosomes, which is more common in males^51^. Further research is needed to better understand the underlying mechanisms of gender disparities in MM incidence, particularly focusing on the role of sex in MM pathogenesis and genetic factors influenced by gender.

Our study has several limitations. Firstly, the study design was purely descriptive, providing a broad overview of trends in incidence and mortality due to multiple myeloma; however, this study did not specifically investigate the social and structural factors contributing to these disparities. Future studies should incorporate population-level social and behavioral data, including socioeconomic status, diet and nutrition, physical activity level, access to healthcare, health literacy, environmental exposures, employment in high-risk industries, lifestyle factors, chronic diseases, and more granular data on residential status. Secondly, due to the cross-sectional study design, we were unable to establish potentially causal relationships between the observed disparities in incidence and mortality and other sociodemographic factors we examined. Thirdly, given the comprehensive nature of our paper aimed at providing an overview of incidence and mortality, we relied on ecological-level data from SEER and CDC WONDER, omitting analyses on patient-level characteristics and social determinants of health, such as socioeconomic status, access to health services, health literacy, screening, and comorbidities. Fourthly, we were unable to explore trends in the incidence and mortality of established precursors of MM, such as MGUS or SMM, limiting our ability to understand the impact of early health interventions that diagnose MM precursors and enable early treatment on MM rates. Lastly, while we included AAPI data as a single aggregate group with relatively lower incidence and mortality rates compared to other SIRE groups, we lacked disaggregated data for AAPI subgroups, hindering our analysis of disparities between these subgroups. Future studies should strive to investigate associations between trends in MM incidence and mortality with social determinants of health and clinical factors to gain deeper insights into the drivers of spatiotemporal changes and disparities.

Despite significant advancements in oncological care and treatment, MM remains a significant burden on the US healthcare system, with increasing incidence over the past two decades. Non-Hispanic Black Americans, in particular, face disproportionately high rates of MM incidence and mortality, underscoring the imperative for equity-centered MM screening and treatments. Understanding SIRE disparities in MM is crucial for guiding public health efforts aiming to improve access to MM detection, treatment, prognosis, survival, and quality of life for patients with MM. Proactive measures—considering social, genetic, and healthcare access factors—are necessary to reduce MM burden, especially within marginalized communities, requiring comprehensive epidemiological surveillance and targeted interventions.

## Data availabilty

Data collected and analyzed in this study are publicly available on the SEER and CDC WONDER websites.

### Supplementary Information

Supplementary Information.
