# Supplementary material for: Multiple myeloma incidence and mortality trends in the United States, 1999–2020
Source: Sci Rep. 2024 Jun 24;14:14564. doi: 10.1038/s41598-024-65590-4 (PMC11196710; doi:10.1038/s41598-024-65590-4)
Supplement: Supplementary file 1 — Supplementary Information. [file 41598_2024_65590_MOESM1_ESM.docx]

# Supplementary Table 1. Incidence rates for multiple myeloma in 2020, across racial/ethnic groups

| **Demographic and clinical characteristics** | **Non-Hispanic White** | | **Non-Hispanic Black** | | **Non-Hispanic**  **PRCDA/AIAN** | | **Non-Hispanic**  **Asian/Pacific Islander** | | **Hispanic**  **Overall** | |
| --- | --- | --- | --- | --- | --- | --- | --- | --- | --- | --- |
|  | **IR**  (per 100,000) | **IRR** | **IR**  (per 100,000) | **IRR** | **IR**  (per 100,000) | **IRR** | **IR**  (per 100,000) | **IRR** | **IR**  (per 100,000) | **IRR** |
| **Sex** |  |  |  |  |  |  |  |  |  |  |
| Male | 7.84  (7.34, 8.37) | 1.00 (ref) | 15.92  (13.86, 18.18) | 2.03 | 5.26  (2.27, 10.25) | 0.67 | 4.93  (4.16, 5.79) | 0.63 | 7.37  (6.41, 8.42) | 0.94 |
| Female | 5.15  (4.77, 5.55) | 1.00 (ref) | 12.99  (11.41, 14.74) | 2.50 | 6.26  (3.15, 11.01) | 1.22 | 3.21  (2.65, 3.85) | 0.62 | 4.19  (3.57, 4.89) | 0.81 |
| **Age** |  |  |  |  |  |  |  |  |  |  |
| 00-39 years | 0.17  - | 1.00 (ref) | 0.31  - | 1.82 | — | — | 0.21  - | 1.24 | 0.08  - | 0.47 |
| 40-44 years | 1.84  (1.12, 2.83) | 1.00 (ref) | 4.69  (2.34, 8.33) | 2.55 | 4.68  (0.12, 24.16) | 2.54 | 0.48  (0.06, 1.70) | 0.26 | 3.35  (2.10, 5.06) | 1.82 |
| 45-49 years | 3.31  (2.30, 4.59) | 1.00 (ref) | 8.89  (5.43, 13.67) | 2.69 | 0.00  (0.00, 16.09) | 0.00 | 3.21  (1.71, 5.47) | 0.97 | 4.72  (3.16, 6.77) | 1.43 |
| 50-54 years | 5.72  (4.42, 7.27) | 1.00 (ref) | 19.08  (13.81, 25.65) | 3.34 | 5.19  (0.13, 26.82) | 0.91 | 4.37  (2.50, 7.06) | 0.76 | 5.74  (3.93, 8.09) | 1.00 |
| 55-59 years | 11.00  (9.26, 12.97) | 1.00 (ref) | 27.49  (21.07, 35.19) | 2.50 | 19.11  (5.21, 47.51) | 1.74 | 5.94  (3.68, 9.05) | 0.54 | 7.06  (4.89, 9.85) | 0.64 |
| 60-64 years | 16.07  (13.95, 18.41) | 1.00 (ref) | 35.05  (27.27, 44.30) | 2.18 | 27.06  (8.79, 61.63) | 1.68 | 13.04  (9.40, 17.60) | 0.81 | 20.18  (15.98, 25.13) | 1.26 |
| 65-69 years | 24.32  (21.52, 27.37) | 1.00 (ref) | 56.33  (44.87, 69.76) | 2.32 | 34.55  (11.22, 78.68) | 1.42 | 17.27  (12.74, 22.87) | 0.71 | 22.05  (16.90, 28.23) | 0.91 |
| 70-74 years | 32.20  (28.66, 36.04) | 1.00 (ref) | 60.89  (47.09, 77.37) | 1.89 | 19.10  (2.31, 65.69) | 0.59 | 21.06  (15.53, 27.88) | 0.65 | 26.07  (19.47, 34.14) | 0.81 |
| 75-79 years | 39.95  (35.20, 45.14) | 1.00 (ref) | 84.94  (64.50, 109.64) | 2.13 | 14.87  (0.38, 76.84) | 0.37 | 18.42  (12.24, 26.55) | 0.46 | 30.13  (21.42, 41.11) | 0.75 |
| 80-84 years | 50.24  (43.62, 57.55) | 1.00 (ref) | 92.28  (66.22, 124.93) | 1.84 | 49.69  (6.02, 170.84) | 0.99 | 28.13  (18.89, 40.05) | 0.56 | 31.03  (20.45, 45.02) | 0.62 |
| 85+ years | 36.67  (31.35, 42.62) | 1.00 (ref) | 82.18  (56.90, 114.53) | 2.24 | — | — | 13.30  (7.75, 21.20) | 0.36 | 30.67  (20.21, 44.50) | 0.84 |

**Abbreviations:** PRCDA, Purchased/Referred Care Delivery Area; AIAN, American Indian Alaskan Natives; IR, incidence rate; IRR, incidence rate ratios. — indicates missing or suppressed data values due to small sample sizes.

# Supplementary Table 2. Mortality rates for multiple myeloma in 2020, across racial/ethnic groups

| **Demographic and clinical characteristics** | **Non-Hispanic White** | | **Non-Hispanic Black** | | **Non-Hispanic**  **PRCDA/AIAN** | | **Non-Hispanic**  **Asian/Pacific Islander** | | **Hispanic**  **Overall** | |
| --- | --- | --- | --- | --- | --- | --- | --- | --- | --- | --- |
|  | **MR**  (per 100,000) | **MRR** | **MR**  (per 100,000) | **MRR** | **MR**  (per 100,000) | **MRR** | **MR**  (per 100,000) | **MRR** | **MR**  (per 100,000 | **MRR** |
| **Sex** |  |  |  |  |  |  |  |  |  |  |
| Male | 3.64 (3.54, 3.74) | 1.00 (ref) | 7.02 (6.59, 7.45) | 1.93 | 1.98 (1.27, 2.95) | 0.54 | 1.97 (1.68, 2.26) | 0.54 | 3.05 (2.78, 3.32) | 0.84 |
| Female | 2.20 (2.13, 2.27) | 1.00 (ref) | 4.93 (4.65, 5.22) | 2.24 | 1.88 (1.23, 2.76) | 0.85 | 1.13 (0.94, 1.32) | 0.51 | 2.07 (1.88, 2.26) | 0.94 |
| **Age** |  |  |  |  |  |  |  |  |  |  |
| 00-39 years | — | 1.00 (ref) | — | — | — | — | — | — | — | — |
| 40-44 years | — | 1.00 (ref) | — | — | — | — | — | — | — | — |
| 45-49 years | 0.87 (0.70, 1.04) | 1.00 (ref) | 1.97 (1.47, 2.60) | 2.26 | — | — | — | — | 0.80 (0.55, 1.14) | 0.92 |
| 50-54 years | 1.58 (1.37, 1.80) | 1.00 (ref) | 3.54 (2.85, 4.35) | 2.24 | — | — | — | — | 1.24 (0.89, 1.68) | 0.78 |
| 55-59 years | 2.65 (2.38, 2.91) | 1.00 (ref) | 7.34 (6.31, 8.37) | 2.77 | — | — | 1.73 (1.07, 2.65) | 0.65 | 2.56 (2.02, 3.21) | 0.97 |
| 60-64 years | 4.24 (3.91, 4.57) | 1.00 (ref) | 10.86 (9.55, 12.17) | 2.56 | — | — | 3.01 (2.07, 4.23) | 0.71 | 4.72 (3.84, 5.61) | 1.11 |
| 65-69 years | 7.35 (6.89, 7.82) | 1.00 (ref) | 16.32 (14.51, 18.14) | 2.22 | — | — | 3.68 (2.55, 5.14) | 0.50 | 7.30 (6.03, 8.58) | 0.99 |
| 70-74 years | 13.11 (12.44, 13.78) | 1.00 (ref) | 24.62 (22.02, 27.23) | 1.88 | — | — | 7.37 (5.54, 9.62) | 0.56 | 13.32 (11.30, 15.34) | 1.02 |
| 75-79 years | 20.37 (19.36, 21.37) | 1.00 (ref) | 45.33 (40.87, 49.79) | 2.23 | — | — | 10.77 (8.02, 14.16) | 0.53 | 16.34 (13.57, 19.10) | 0.80 |
| 80-84 years | 30.23 (28.71, 31.75) | 1.00 (ref) | 54.66 (48.54, 60.79) | 1.81 | — | — | 16.78 (12.57, 21.95) | 0.56 | 27.54 (23.15, 31.93) | 0.91 |
| 85+ years | 38.11 (36.43, 39.78) | 1.00 (ref) | 66.14 (59.18, 73.10) | 1.74 | — | — | 16.49 (12.28, 21.68) | 0.43 | 28.05 (23.56,32.54) | 0.74 |
| **US census region** |  |  |  |  |  |  |  |  |  |  |
| Northeast | 3.27 (3.11, 3.44) | 1.00 (ref) | 6.12 (5.45, 6.79) | 1.87 | — | — | 1.67 (1.24, 2.21) | 0.51 | 2.22 (1.84, 2.61) | 0.68 |
| West | 3.85 (3.68, 4.03) | 1.00 (ref) | 6.85 (5.89, 7.81) | 1.78 | — | — | 1.94 (1.65, 2.24) | 0.50 | 2.70 (2.43, 2.97) | 0.70 |
| South | 3.48 (3.36, 3.61) | 1.00 (ref) | 7.72 (7.30, 8.14) | 2.22 | 2.82 (1.74, 4.31) | 0.81 | 1.97 (1.51, 2.52) | 0.57 | 2.28 (2.04, 2.52) | 0.66 |
| Midwest | 3.84 (3.68, 4.00) | 1.00 (ref) | 7.53 (6.77, 8.28) | 1.96 | — | — | 2.17 (1.47, 3.08) | 0.57 | 3.10 (2.44, 3.90) | 0.81 |
| **Urbanization** |  |  |  |  |  |  |  |  |  |  |
| Large central metro | 3.42 (3.26, 3.58) | 1.00 (ref) | 6.94 (6.48, 7.39) | 2.28 | — | — | 1.8 (1.52, 2.08) | 0.59 | 2.60 (2.37, 2.83) | 0.85 |
| Large fringe metro | 3.50 (3.35, 3.65) | 1.00 (ref) | 7.61 (6.96, 8.27) | 2.54 | — | — | 2.03 (1.61, 2.53) | 0.68 | 2.13 (1.79, 2.47) | 0.71 |
| Medium metro | 3.70 (3.54, 3.86) | 1.00 (ref) | 7.74 (6.99, 8.49) | 2.47 | — | — | 2.08 (1.56, 2.72) | 0.66 | 2.65 (2.28, 3.02) | 0.85 |
| Small metro | 3.70 (3.47, 3.93) | 1.00 (ref) | 8.10 (6.83, 9.37) | 2.59 | — | — | — | — | 2.47 (1.82, 3.27) | 0.79 |
| Micropolitan (nonmetro) | 3.79 (3.55, 4.03) | 1.00 (ref) | 7.78 (6.42, 9.13) | 2.49 | — | — | — | — | 2.18 (1.54, 3.01) | 0.70 |
| Noncore (nonmetro) | 3.67 (3.41, 3.94) | 1.00 (ref) | 6.79 (5.44, 8.14) | 2.25 | — | — | — | — | — | — |

**Abbreviations:** AIAN, American Indian Alaskan Natives; AAPI, Asian American Pacific Islanders; MR, mortality rate; MRR, mortality rate ratios. — indicates missing or suppressed data values due to small sample sizes.
